# Human amniotic stem cells-derived exosmal miR-181a-5p and miR-199a inhibit melanogenesis and promote melanosome degradation in skin hyperpigmentation, respectively

**DOI:** 10.1186/s13287-021-02570-9

**Published:** 2021-09-10

**Authors:** Xiao-Yu Wang, Xiao-Hui Guan, Zhen-Ping Yu, Jie Wu, Qi-Ming Huang, Ke-Yu Deng, Hong-Bo Xin

**Affiliations:** 1 The National Engineering Research Center for Bioengineering Drugs and the Technologies, Institute of Translational Medicine, Nanchang University, Nanchang, 330031 Jiangxi China; 2 College of Life Science, Nanchang University, 999 Xuefu Road, Honggutan District, Nanchang, 330031 Jiangxi China

**Keywords:** Human amniotic stem cells, Exosomes, Autophagy, Hyperpigmentation, miRNA

## Abstract

**Background:**

Hyperpigmentation of skin is caused by an imbalance between the melanosome/melanin synthesis in melanocytes and the melanosome/melanin degradation in keratinocytes. Although studies showed that stem cells play a role in hypopigmentation, the underlying mechanisms are far not elucidated. Human amniotic stem cells (hASCs) including human amniotic mesenchymal stem cells (hAMSCs) and human amniotic epithelial stem cells (hAESCs) were considered to be a promising cell source for stem cells-based therapy of many diseases clinically due to their pluripotent potential, no tumorigenesis and immunogenicity, no ethical issues, and potent paracrine effects. Here, we reported that both hASCs and their conditional medium (CM) had a potent anti-hyperpigmentation in skin in vivo and in vitro.

**Methods:**

hAESCs and hAMSCs were identified by RT-PCR, flow cytometric analysis and immunofluorescence. Effects of hASCs and hASC-CM on pigmentation were evaluated in B16F10 cells stimulated with α-melanocyte-stimulating hormone (α-MSH), and mouse ears or human skin substitutes treated with ultraviolet radiation B (UVB). Expressions of the key proteins related with melanogenesis and autophagic flux were detected by western blot in B16F10 cells for further exploring the effects and the underlying mechanisms of hAESC-CM and hAMSC-CM on melanogenesis and melanosome degradation. The hAMSCs exosomes-derived miRNAs were determined by sequencing. RT-PCR, western blot, melanin content analysis and luciferase activity assay were used to determine the hypopigmentation of miR-181a-5p and miR-199a.

**Results:**

In our study, we observed that both hASCs and their CM significantly alleviated the α-MSH in B16F10 cells or UVB-induced hyperpigmentation in mouse ears or human skin substitutes by suppressing melanin synthesis and promoting melanosome degradation in vivo and in vitro. Furthermore, we demonstrated that miR-181a-5p and miR-199a derived from hASCs exosomes remarkably inhibited melanogenesis by suppressing MITF (microphthalmia-associated transcription factor) which is a master regulator for governing melanogenesis and promoting melanosome degradation through activating autophagy, respectively.

**Conclusions:**

Our studies provided strong evidence that the conditional medium and exosomes derived from hAMSCs inhibit skin hyperpigmentation by suppressing melanogenesis and promoting melanosome degradation, indicating that the hASCs exosomes or their released microRNAs might be as reagents for cell-free therapy in hyperpigmented disorders clinically.

**Supplementary Information:**

The online version contains supplementary material available at 10.1186/s13287-021-02570-9.

## Background

Skin pigmentation is an intricate multistep process leading to the distribution of melanin particles throughout the interaction between two epidermal cell types: keratinocytes and melanocytes [1, 2]. Melanin is produced in a specific organelle, called melanosome, which is formed in the melanocytes of the skin [3]. The melanosomes are transported from the perinuclear region to the dendritic tips of melanocytes and are subsequently transferred into neighboring keratinocytes during the process of skin color development [4, 5]. UV-exposed keratinocytes secrete α-MSH, which binds to the melanocortin1 receptor (MC1R) on the surface of melanocytes to activate cAMP cascades and induces MITF expression [6, 7]. MITF is a master regulator for governing melanogenesis and the expression of many genes which are involved in melanocyte development and function [8], in which the core proteins for melanin biosynthesis include tyrosinase (Tyr) and tyrosinase-related protein 1 (TRP1) [9]. During this complex process, the total amount of melanosomes has a considerable impact on skin color.

Melanosome/melanin degradation represents another way for determining skin color, which was dependent on the rates of melanin formation in melanocytes and transference from melanocytes to keratinocytes. It has been reported that autophagy induction regulated skin pigmentation by causing melanosome degradation in keratinocytes and melanocytes, especially excessive pigment deposition [10–13]. Autophagy delivers cytoplasmic cargo to the lysosome to form an autolysosome for degradation through the intermediary of a double membrane-bound vesicle, referred to as an autophagosome [14]. As autophagy targets organelles such as mitochondria, peroxisomes, and the endoplasmic reticulum, it would be reasonable to speculate that melanosome degradation might be mediated by autophagy. Therefore, if we are able to find some agents that not only inhibit melanin synthesis, but also promote melanosomes degradation, may be beneficial for treatment of hyperpigmentation.

Recently, stem cell-based therapies hold the potential to alleviate the burden of many serious diseases [15]. Especially, mesenchymal stem cells (MSCs) promote cutaneous wound healing and skin regeneration by accelerating wound closure, enhancing reepithelialization, increasing angiogenesis, modulating inflammation, and regulating ECM remodelling [16–18]. Numerous studies indicated that MSCs-exosome which is a membranous nanovesicle with 30–100 nm in size and formed from endosomal compartment, might be as a novel reagent of the cell-free therapy for cutaneous regeneration. A breakthrough in exosome research was the finding of their nucleic acid contents, such as messenger RNA, microRNA, and mitochondrial DNA, which could be transported to other cells [19, 20]. Exosome delivery system is an attractive model for clinical application without any side effect. Especially, the miRNAs in exosomes were able to serve as the mediators for paracrine and endocrine communication between different tissues, modulating gene expressions and the function of distal cells. It is demonstrated that miRNAs bind to the base pair with the 3′-untranslated region (3’UTR) of target mRNAs for subsequently inhibiting protein synthesis by either repressing translation or promoting mRNA degradation [21], emerging as new therapeutic agents and diagnostic makers for a range of disorders.

Studies showed that hASCs including hAMSCs and hAESCs were considered to be a promising cell source for potential clinical application due to the active proliferative potential, no immunogenicity and tumorigenicity, no etheric issue, anti-inflammatory function, and tissue repair ability, especially in skin wound repair [22, 23]. Recently, we reported that hAMSCs and their paracrine factors promoted thermal burn wound healing [24]. However, the effect of hASCs and their conditional medium on skin hyperpigmentation is not explored.

In the present study, we observed that hASCs and their conditional medium significantly inhibited α-MSH or UVB-induced hyperpigmentation by suppressing melanogenesis and promoting melanosome degradation in vivo and in vitro. We further observed that the effects of hASCs and their conditional medium on skin pigmentation were reversed by the exosome inhibitor [25], suggesting that hASCs-derived exosomes may play a key role in their inhibition of skin hyperpigmentation. Subsequently, we analyzed the exosomal miRNA expression profiles by deep sequencing and we further demonstrated that the miR-181a-5p and miR-199a derived from hASCs exosomes remarkably inhibited melanogenesis by suppressing MITF and promoted melanosome degradation through activating autophagy, respectively.

## Methods

### Materials

Keratinocytes HaCat cells were purchased from ATCC and cultured in DMEM supplemented with 15% fetal bovine serum (FBS, Thermo Fisher Scientific, USA). B16F10 cells from National Infrastructure of Cell Line Resource were cultured in DMEM (Thermo Fisher Scientific, USA) containing 10% FBS. 3D-HSSs (MelaKutis®) was purchased from Guangdong Biocell Biotechnology Company (Guangdong, China). Adult male C57BL/6 mice (8 weeks old) were purchased from Changsha SLAC Laboratory Animal Company (Changsha, China, http://www.hnsja.com/). Animal procedures were conducted according to the Laboratory Animal Center of Institute of Translational Medicine of Nanchang University Guidelines and reviewed and approved by the Animal Care and Use Committee of Nanchang University, and conformed to the Guide for the Care and Use of Laboratory Animals published by the US National Institutes of Health. PKH26 red fluorescent cell linker mini kit (Sigma, USA), Bafilomycin A1 (APExBIO, USA), TRIzol reagent (Gibco, USA) and the cDNA synthesis kit (Thermo Fisher Scientific, USA), and α-Melanocyte-stimulating hormone (α-MSH) and GW4869 (MCE, China) were purchased from the suppliers.

### Isolation and culture of hAMSCs and hAESCs

Human placental tissues after eutocia or caesarean sections were collected from the Department of Obstetrics and Gynecology, the First Affiliated Hospital of Nanchang University for isolation of human amniotic membrane. This study was approved by the Ethics Committee of the First Affiliated Hospital of Nanchang University. The obtained tissue samples were only used for scientific research. Informed consent was obtained from all of the patients who voluntarily donated their placentas prior to their participation.

hAESCs: Primary cell culture was performed as previously described. Amnions were mechanically separated from the chorion and washed with D-Hank's supplemented with 100 U/ml penicillin and streptomycin. Amnions were then incubated with 0.25% trypsin solution at 37 ℃ for 45 min. Supernatants were collected and centrifuged at 1,500 rpm for 5 min. Then, the cells were seeded in 100 mm culture dishes in DMEM/F12 (Gibco, USA) supplemented with 10% FBS, 200 mM L-glutamine (Gibco, USA), 1% NEAA (Gibco, USA), 1% penicillin/streptomycin, 1‰ β-Mercaptoethanol (Gibco, USA) and 50 ng/mL EGF [26].

hAMSCs: The amnion tissue was then digested with collagenase IV (1 g/L, Worthington, USA) on a rotator at 37 °C for 40 min. The medium containing 10% FBS was then added to terminate digestion, and supernatants were filtered through a 70 µm cell strainer and centrifuged for 3 min at 1000 rpm. The cells were seeded with α-MEM medium (Gibco, USA) containing 10% FBS, 1% glutamine (Gibco, USA), 18% Chang B (Irvine Scientific, USA), 2% Chang C (Irvine Scientific, USA), and 1% penicillin/streptomycin [27].

### Collection of conditioned medium (CM) of hAMSCs or hAESCs

For the collection of hAMSC-CM or hAESC-CM, hAMSCs or hAESCs were grown in a normal culture medium. Once the cells reached 90% confluence, the medium was changed to DMEM without FBS. CM was collected after 48 h and centrifuged at 1500 rpm for 5 min to remove cellular debris. CM was then concentrated by using an Amicon® Ultra 3 K device (Millipore, USA). Dilute CM with B16F10/HaCat cell culture medium to the desired concentration. The final concentration of hASC-CM was defined as a concentration which was equivalent to the conditional medium collected from certain number of cells cultured in serum-free medium for 48 h.

### Identification of hAMSCs and hAESCs by flow cytometry

Flow cytometry was used to identify the characteristics of the cultured hAMSCs and hAESCs and detect related cell surface markers. Passage 3 hAMSCs and hAESCs (1 × 10^6^ cells/mL) were collected and incubated with FITC-conjugated antibodies against human CD29, CD90, CD45, HLA-DR, CD80, and CD40; phycoerythrin (PE)-conjugated antibodies against human CD73, CD105, CD34 and HLA-ABC (all from BD Biosciences, USA) at 4 °C for 30 min in the dark. Appropriate isotype-matched antibodies were used as negative controls. Data from 10,000 viable cells were acquired by a FACSCalibur instrument and analyzed by FCS Express software.

### Melanin content analysis and tyrosinase activity assay

Melanin content was investigated according to a previously published protocol. For the melanin secretion assay, absorbance of culture medium was measured at 475 nm by using an optical density reader. The B16F10 cells were collected, washed twice with ice-cold phosphate-buffered saline (PBS), and centrifuged at 12,000 rpm for 15 min. The pellets were dissolved in dissolving buffer (1 M NaOH, 10% DMSO) for 30 min at 56 °C and solubilized melanin was measured at 405 nm [28].

B16F10 cells were treated with 200 nM α-MSH and hAESC-CM or hAMSC-CM for 48 h. A total of 50 μL cell lysate and 50 μL of 2 mM L-DOPA was mixed with mushroom tyrosinase (100 U/mL) in a well of a 96-well plate for 15 min at room temperature. After incubation, absorbance values were recorded at 475 nm using an optical density reader (SpectraMax M5, USA).

### In vitro co-culture experiment

In the co-culture experiment, a co-culture transwell chamber (2.4-cm diameter, 0.4-μm pore size, Corning, USA) was used to assess the effects of hAMSCs or hAESCs on melanin content in vitro. B16F10 cells were trypsinized and seeded in the lower chamber at a density of 1.5 × 10^5^ cells/well in 2.0 mL of DMEM with 10% FBS, and hAMSCs or hAESCs were seeded in the upper compartment in 1.0 mL medium. After incubation for 48 h, samples were collected and subjected to melanin content analysis as described below.

### Exosome extraction

Exosomes were isolated from the 100 mL hAMSC or hAESC conditional medium by successive differential centrifugation steps at 1000*g* for 20 min and 12,000*g* for 30 min at 4 °C. The supernatant was filtered through a 0.22-μm filter and ultracentrifuged at 100,000*g* for 2 h at 4 °C (Beckman, USA). The morphology of exosomes was observed under transmission electron microscope (Hitachi, Japan).

### Real-time PCR and western blot analysis

Total RNA was extracted using TRIzol total RNA isolation reagent, according to the manufacturer’s instructions. miRNAs were detected with stem-loop primers purchased from RiboBio (Guangzhou, China). GAPDH and U6 small nucleolar RNA was used for normalization. qPCR was performed using the FastStart Universal SYBR Green Master (ROX) on a Real-Time PCR System (Applied Biosystems, USA). Sequences of Real-Time PCR primers are listed in Additional file 1: Table S1.

After treatment with hAESC-CM or hAMSC-CM for 48 h, B16F10 cells were lysed in RIPA buffer, containing protease inhibitor cocktail and total proteins were subjected to 10% or 12% SDS-PAGE, then transferred to PVDF membranes, which were incubated with primary antibodies anti-β-actin, anti-LC3B, anti-P62 (CST, USA), anti-TRP1, anti-MITF (Sigma, USA), anti-PCNA (OriGene, USA), anti-AKT, anti-P-AKT, anti-β-Catenin, anti-GSK3β, anti-p-GSK-3β (Abcam, USA) at 4 °C overnight. The membranes were incubated for 1 h at room temperature with horseradish peroxidase (HRP)-conjugated anti-mouse or anti-rabbit secondary antibodies. Images were quantified using a digital gel image analysis system TANON 5500 (Shanghai, China) and the band intensities were quantified by Tanon GIS software.

### UV exposure

Male C57BL/6 mice with age of 8–10 weeks were exposed to ultraviolet irradiation in UV chamber equipped with 20 W UVB bulbs (Philips, Netherlands) at a dose of 150 mJ/cm^2^. UV emittance was measured with the use of an Ultraviolet detector (Tenmars, China). After 7 days, hAMSC-CM was injected into the lower edge of ears, followed by another 7 days of UVB irradiation. The mice were killed and skin samples were fixed in 4% paraformaldehyde on the 14th day of UVB exposure.

### Histology

Skin tissue were collected and fixed in 4% paraformaldehyde, embedded in paraffin, and serially sectioned at a thickness of 5 μm. The samples were stained with LC3B antibody and was observed under light microscopy (Olympus, Japan).

### Measurement of skin color

The intensity of pigmentation in 3D-HSSs was measured by using a colorimeter (DSM II, Denmark) and were expressed as the L* values.

### Fontana–Masson staining

The tissues were fixed with 10% buffered formalin, and then embedded in paraffin. Melanin pigment was directly detected by using Fontana–Masson staining with an eosin counterstaining (Solarbio, China).

### Small RNA library preparation and sequencing

miRNA sequencing in hAMSCs-exo was carried out by LC-Bio Technology CO., Ltd., Hangzhou, China. Small RNA library was prepared with TruSeq Small RNA Sample Prep Kits (Illumina, San Diego, USA) and the sequencing was performed using Illumina Hiseq2000/2500.

### Luciferase activity assay

293 T cells were seeded at 2 × 10 ^4^ cells per well in 96-well solid white polystyrene microplates (Corning, USA). After 16 h, cells were co-transfected with miR-181a-5p mimic and pmirGLO-WT-3’UTR or pmirGLO-MUT-3’UTR using Lipofectamine 3000 (Thermo Fisher Scientific, USA) for 48 h according to the manufacturer’s instructions. The luciferase activity was detected with the Dual-Luciferase Reporter Assay System (Promega, USA). The relative levels of luciferase activity were normalized to the constitutively expressed Renilla.

### Statistical analysis

The results were analyzed by paired *t*-test, or analysis of variance. *p* value < 0.05 was considered to be statistically significant. Data from three separate experiments are presented as mean ± SD.

## Results

### Identification and characterization of hAESCs or hAMSCs

**Fig. 1:**
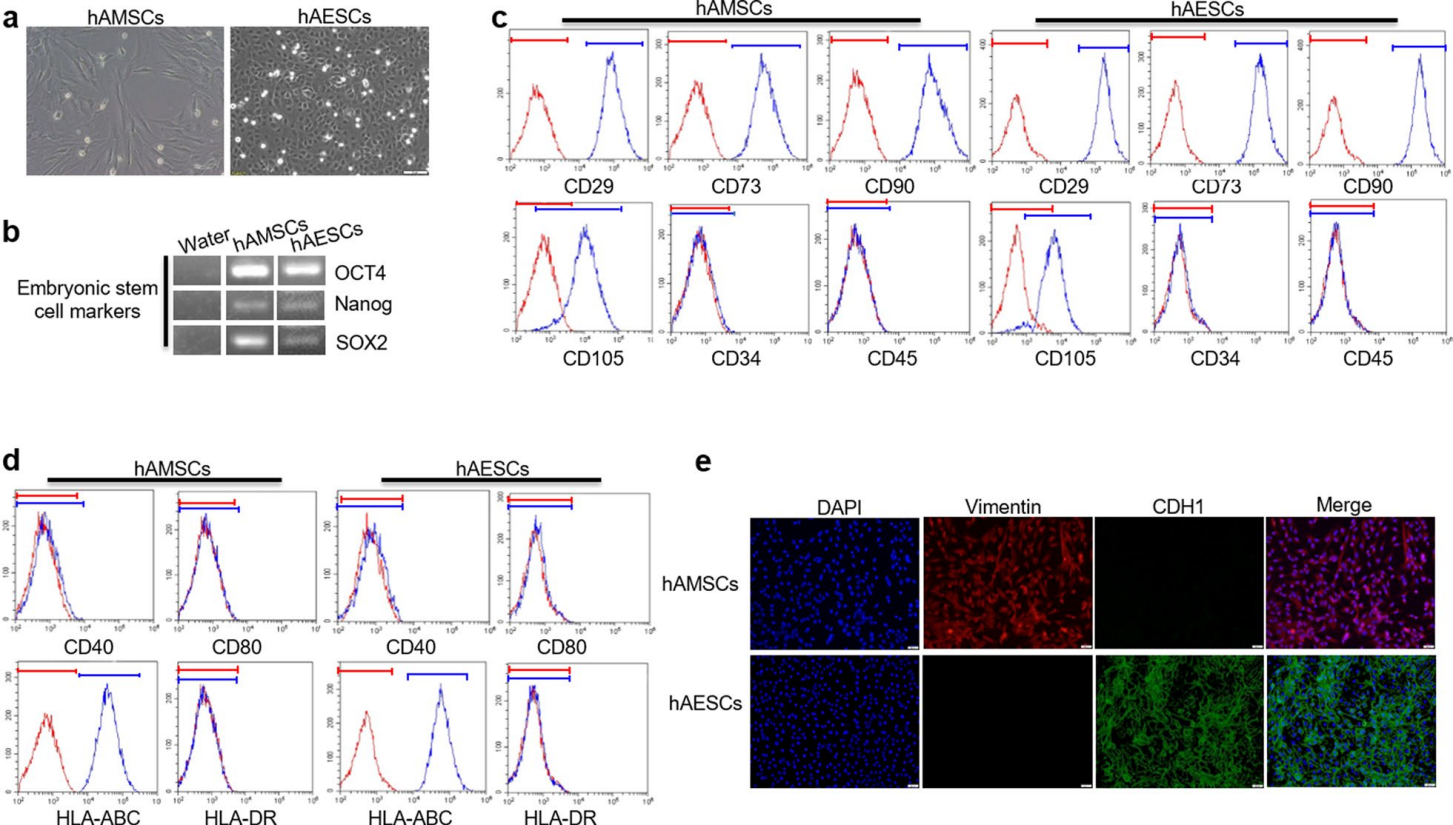
Identification of hAMSCs and hAESCs. **a** Representative photomicrograph of adherent hAMSCs and hAESCs. Scale bar = 50 μm. **b** The expressions of markers including Nanog, SOX2 and OCT4 in hAMSCs or hAESCs were analyzed by RT-PCR and water was used as negative control. **c** The expressions of markers including CD29, CD90, CD73, CD105, CD34, CD45 were analyzed by flow cytometry in hAMSCs or hAESCs. The red lines represent the isotype control, and the blue lines represent the surface markers. **d** The expressions of markers including HLA-DR, CD80, CD40 and HLA-ABC were analyzed by flow cytometry in hAMSCs or hAESCs. **e** The expressions of vimentin and CDH1 were detected by immunofluorescence staining in hAMSCs and hAESCs. Scale bar = 20 μm. Data are presented as mean ± SD. The experiments were repeated three times independently and the data of one representative experiment was shown

hASCs including hAESCs and hAMSCs were isolated from the amniotic membranes as previously reports. As showed Fig. 1a, hAESCs exhibited a cobblestone-like morphology, and hAMSCs showed a spindle-shaped, fibroblast-like morphology. Both hAESCs and hAMSCs expressed the embryonic/pluripotent stem cell markers such as OCT4, Nanog and SOX2 (Fig. 1b) and the results from flow cytometric analysis showed that hAMSCs and hAESCs were positive for mesenchymal stem cell markers such as CD29, CD73, CD90 and CD105, but were negative for hematopoietic stem cell markers CD34 and CD45 (Fig. 1c). Most importantly, although hAMSCs and hAESCs expressed the major histocompatibility protein HLA-ABC, there were no expressions of the co-stimulatory molecules such as CD40 and CD80 and the major histocompatibility protein HLA-DR (Fig. 1d), indicating that the cells possess a low immunogenicity. In addition, hAESCs and hAMSCs were further confirmed by applying the specific antibodies against the epithelial marker CDH1 or mesenchymal marker vimentin, respectively, and the results showed that more than 90% of hAMSCs expressed vimentin, whereas most of hAESCs expressed CDH1 (Fig. 1e). Then, we identified the multipotency of hAMSCs by osteogenic and chondrogenic differentiation assays (Additional file 1: Fig. S1a).

Next, we investigated the effect of the CM obtained from the cultured hAMSCs and hAESCs on the proliferation and migration of B16F10 and the results showed that both hAMSC-CM and hAESC-CM did not affect the proliferation (Additional file 1: Fig. S1b) and migration (Additional file 1: Fig. S1c) of B16F10 at 2 × 10^5^ cells/mL for 2 days, suggesting there was no cytotoxicity on B16F10 cells treated with hAMSC-CM or hAESC-CM.

### hAMSC-CM or hAESC-CM inhibited α-MSH-induced melanogenesis through suppressing MITF in B16F10 cells

**Fig. 2:**
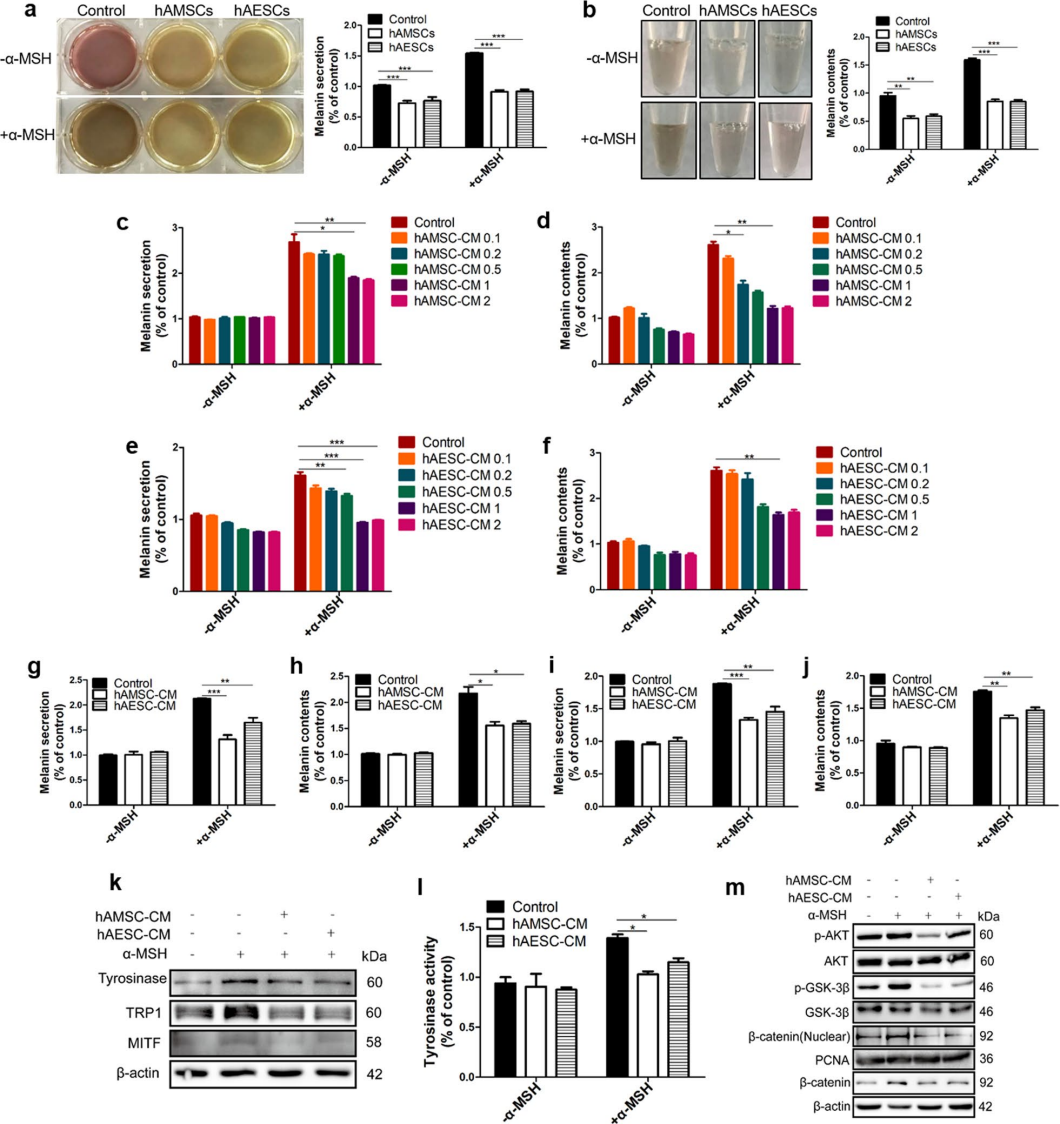
hAMSC-CM or hAESC-CM inhibits α-MSH-induced melanogenesis in B16F10 cells. hAMSCs or hAESCs were co-cultured with B16F10 for 2 days and melanin pigments were quantified by measuring absorbance values of cell supernatant (**a**) and cell precipitation suspension (**b**). B16F10 cells were stimulated with α-MSH and then treated with various concentrations of hAMSC-CM or hAESC-CM (concentrations from 2 × 10^4^ to 2 × 10^6^ cells/mL, and hAMSC-CM 1 or hAESC-CM 1 indicates the concentration of 2 × 10^5^ cells/mL) for 48 h, the cell supernatants (**c**, **e**) and cell pellets (**d**, **f**) were obtained to determine anti-melanogenesis effect of hAMSC-CM (**c**, **d**) or hAESC-CM (**e**, **f**). B16F10 cells were pre-treated with α-MSH (200 nM) for 6 h and then further co-incubated with hAMSC-CM or hAESC-CM for 48 h. The cell supernatants (**g**) and cell pellets (**h**) were obtained for determining the therapeutic effects of hAMSC-CM or hAESC-CM as described in the materials and methods. B16F10 cells were pre-treated with hAMSC-CM or hAESC-CM for 6 h, then α-MSH was added to further incubate for 48 h, and the cell supernatants (**i**) and cell pellets (**j**) were obtained for determining protective effects of hAMSC-CM or hAESC-CM. The effects of hAMSC-CM or hAESC-CM on the protein expression levels of melanogenesis-related protein (tyrosinase, TRP1 and MITF) were analyzed by western blotting analysis (**k**). The effects of hAMSC-CM or hAESC-CM on tyrosinase enzyme activity were confirmed by treatment with L-DOPA and detection of tyrosinase at 475 nm (**l**). The phospho-AKT, phospho-Gsk3β, β-catenin and β-catenin (nuclear) were determined by western blot analysis in B16F10 cells with α-MSH stimulation (**m**). Data are presented as mean ± SD. The experiments were repeated three times independently and the data of one representative experiment was shown. *p < 0.05; **p < 0.01, ***p < 0.001

To examine the effects of hAMSCs and hAESCs on skin pigmentation, B16F10 cells were co-cultured with hAMSCs or hAESCs with or without α-MSH stimulation. The results showed that both hAMSCs and hAESCs significantly alleviated α-MSH-induced the generation and secretion of melanin in B16F10 cells compared with control, and a visual hypopigmentation was observed in both the culture medium (Fig. 2a) and the lysate (Fig. 2b) of B16F10 cells. In addition, α-MSH-induced the secretion and generation of melanin were remarkably inhibited by both hAMSC-CM (Fig. 2c, d) and hAESC-CM (Fig. 2e, f) in B16F10 cells in a concentration-dependent manner. Furthermore, both therapeutic (Fig. 2g, h) and preventative (Fig. 2i, j) administrations of hAMSC-CM or hAESC-CM inhibited cellular melanin content by 52% and 50%, and inhibited melanin secretion by 69% and 42%, respectively, with a concentration of 2 × 10^5^ cells/mL. The observation that both human amniotic stem cells and their conditional medium had a similar efficacy in inhibiting α-MSH-induced the generation and secretion of melanin in B16F10 cells suggested that the hypopigmentation of the stem cells was mediated by their paracrine effect.

Next, we investigated the activities of enzymes involved in melanin production with mushroom tyrosinase. As shown in Fig. 2l, tyrosinase activity was markedly suppressed by hAMSC-CM or hAESC-CM. The western blot analysis also showed that α-MSH-induced expressions of key melanogenesis regulating proteins such as tyrosinase, TRP1 and MITF were significantly inhibited by hAMSC-CM or hAESC-CM in B16F10 cells (Fig. 2k). Previous work indicated that there was a positive regulatory loop by which increased MITF would cause an expansion of late endolysosomal vesicles that potentiates Wnt signaling, in turn, Wnt signaling stabilizes MITF. Similar results were also obtained when B16F10 cells were treated with hAMSC-CM or hAESC-CM, indicating that the hypopigmentation of hASCs-derived CM was associated with inhibition of Wnt signaling (Fig. 2m). Taken together, these results indicated that hAMSC-CM or hAESC-CM-mediated hypopigmentation may be involved in inhibition of the melanogenesis through suppressing MITF-mediated Wnt signaling pathway in B16F10 cells stimulated with α-MSH.

### hAMSC-CM or hAESC-CM-mediated autophagy was required for melanosome degradation

**Fig. 3:**
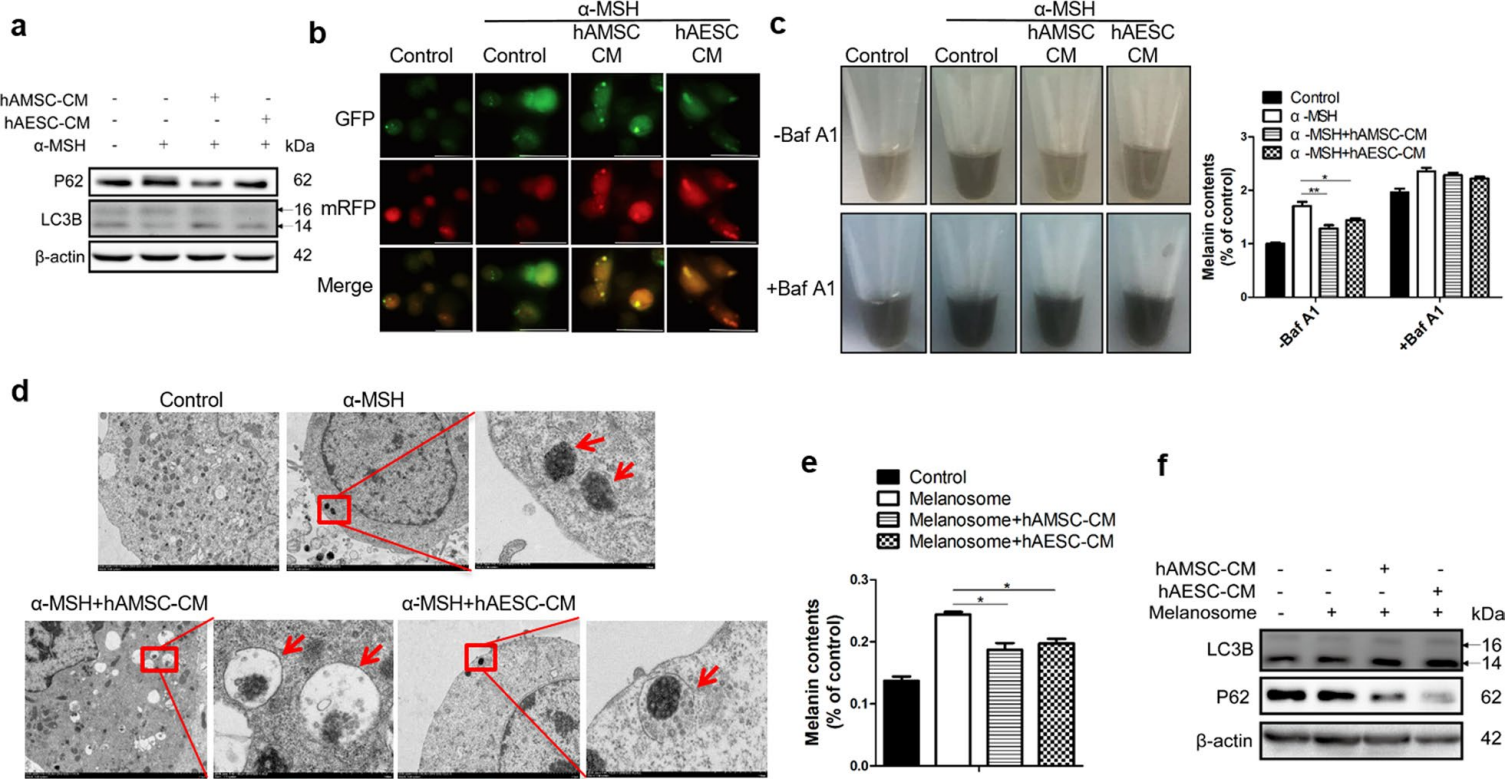
Autophagy was required for the anti-melanogenesis effect of hAMSC-CM or hAESC-CM. B16F10 cells were exposed to hAMSC-CM or hAESC-CM and then the lysates were collected for western-blotting analysis using LC3- and p62-specific antibodies and β-Actin was used as a loading control (**a**). B16F10 cells stably expressing dual-fluorescence mRFP-GFP-LC3B were treated with hAMSC-CM or hAESC-CM for 48 h and then analyzed by fluorescence microscopy, and the red and yellow puncta were counted under 40 × magnification. Scale bar = 20 μm (**b**). B16F10 cells stimulated with α-MSH were incubated with hAMSC-CM or hAESC-CM. The melanin content was analyzed by measuring absorbance in the presence or absence of bafilomycin A1 (**c**). B16F10 cells pre-treated with α-MSH were exposed to hAMSC-CM or hAESC-CM for 48 h and observed with electron microscopy (scale bar = 2 μm), and the red arrows indicate over-produced melanin (only α-MSH group) and autophagosomes that contain melanin or melanosome (hAMSC-CM or hAESC-CM groups) in the higher magnification electron microscopic picture (scale bar = 500 nm) (**d**). Melanosomes were added to the HaCat cells, followed by incubation for 24 h. After washing with PBS, the cells were incubated with hAMSC-CM or hAESC-CM for an additional 48 h, then the cells were collected and lysed for melanin content assay (**e**) and anti-LC3B, anti-P62 antibodies (**f**). The protein intensity was calculated from normalizing against β-actin. Data are presented as mean ± SD. The experiments were repeated three times independently and the data of one representative experiment was shown. *p < 0.05; **p < 0.01, ***p < 0.001

Melanin is synthesized in melanosomes, a lysosome-related organelle in epidermal melanocytes. Moreover, autophagy possesses housekeeping function by both removing misfolded or aggregated proteins and clearing damaged or unnecessary organelles in a lysosome-dependent manner [29]. Accordingly, we postulated that autophagy might play a role in hAMSC-CM or hAESC-CM-mediated the hypopigmentation by promoting melanosome degradation. Thus, we examined whether hAMSC-CM or hAESC-CM influences autophagy. As showed in Fig. 3a, hAMSC-CM or hAESC-CM remarkably increased the conversion of LC3I to LC3II, a marker of autophagosomes, accompanied by degradation of p62 protein. Since autophagy is a highly dynamic process that is responsible for autophagosome synthesis, delivery of autophagic substrates to the lysosomes, and degradation of autophagic substrates inside lysosomes, LC3 measurements were not be able to distinguish from activation or impairment of the autophagy flux. To complement our results, B16F10 cells expressing mRFP-GFP-LC3 were constructed to monitor autophagosomes by observing punctate structures. Here, a yellow fluorescence (due to merged mRFP and GFP) represents that the fusion protein is in a neutral pH environment, whereas a red fluorescence (due to quenching of GFP) implies that the protein has entered acidic lysosomes. As shown in Fig. 3b, a red and merged yellow LC3B puncta was dramatically increased in hAMSC-CM or hAESC-CM-treated cells, suggesting that the activation of autophagic flux was accompanied with the increased autolysosomes.

Next, we further investigated whether the melanosome degradation was the consequence of increased autophagic flux in hAMSC-CM or hAESC-CM-treated cells. The results showed that hAMSC-CM or hAESC-CM markedly inhibited the cellular melanin contents of B16F10 cells compared with the control group, while the suppression was significantly reversed by bafilomycin A1, an inhibitor of autophagosome-lysosome fusion and lysosomal acidification (Fig. 3c). To validate our finding, the cellular autophagosomes and melanosomes were further confirmed by electron microscopy (EM). The results showed that autophagosomes, specialized structures surrounded by two distinct lipid bilayers, were observed in the cells undergoing autophagy with EM and B16F10 cells only treated with α-MSH for 48 h showed the accumulation of melanin, additional treatment with hAMSC-CM or hAESC-CM indicated that the autophagosomes engulfed melanin or melanosomes (Fig. 3d). Collectively, these results suggest that autophagy plays a vital role in hAMSC-CM or hAESC-CM-induced melanosome degradation.

As keratinocytes are the final destination of melanosomes, the total amount of melanosomes in keratinocytes has a considerable impact on skin color [30]. To determine whether hAMSC-CM or hAESC-CM affects the melanosome degradation by autophagy in keratinocytes, the HaCat cells were incubated with melanosome fraction isolated from B16F10 cells in the presence or absence of hAMSC-CM or hAESC-CM. Consistent with the results of B16F10 cells, both hAMSC-CM or hAESC-CM markedly degraded the extrinsic melanosomes (Fig. 3e). Most importantly, the increase of LC3II protein and the decrease of P62 protein expression were observed in HaCat cells treated with hAMSC-CM or hAESC-CM (Fig. 3f). Taken together, these data demonstrated that hAMSC-CM or hAESC-CM promotes melanosome degradation by the activation of autophagy.

### hAMSC-CM alleviated UVB-induced hyperpigmentation in mouse and human skin substitutes

**Fig. 4:**
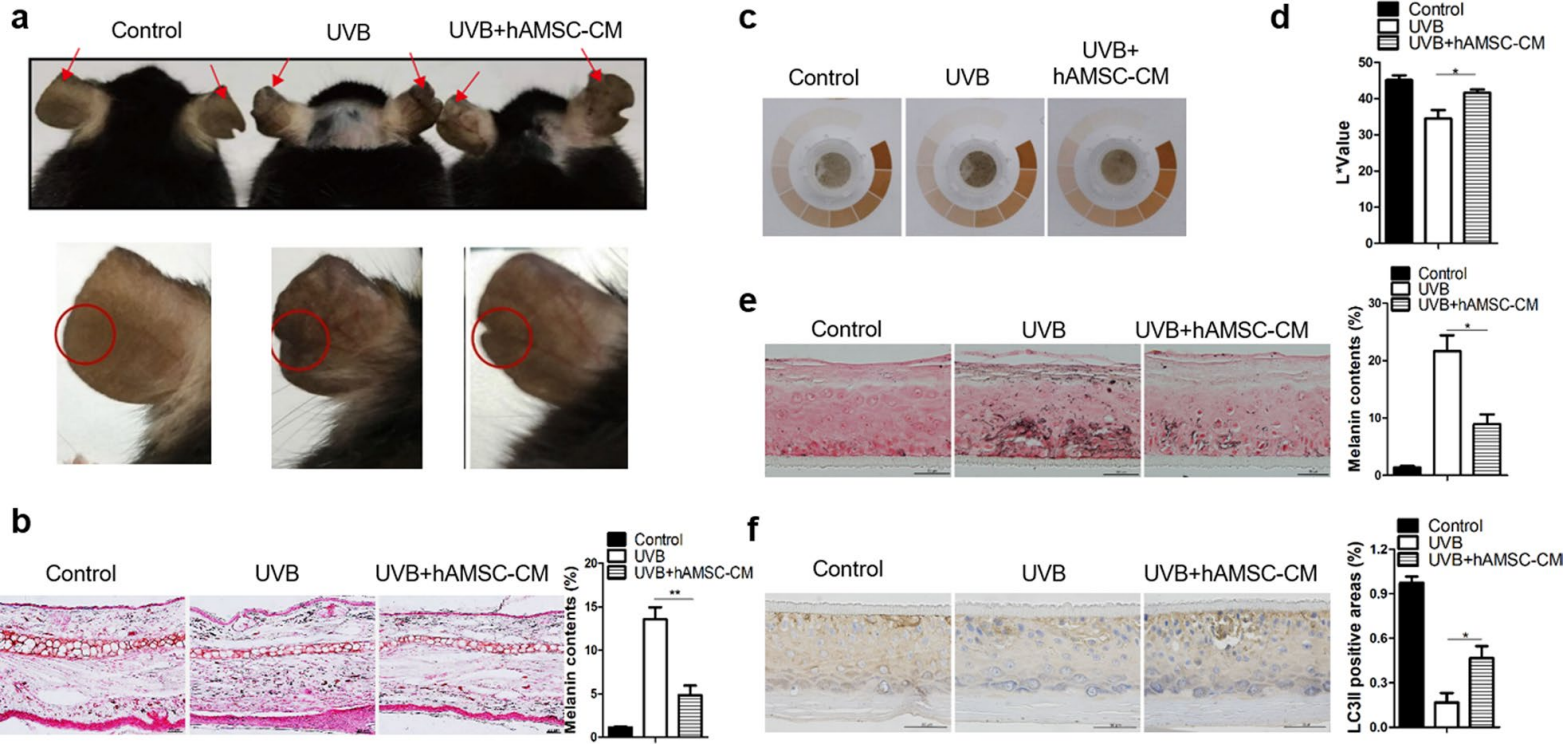
hAMSC-CM inhibited the UVB-induced melanin productions by modulating autophagy in vivo. **a** C57BL/6 mice were irradiated with UVB at a dose of 150 mJ/cm^2^ for 14 days. hAMSC-CM was injected into the lower edge of ears at the eighth day. Arrows indicate pigmentation differences of ear skin with or without hAMSC-CM treatment. Epidermis of ear skin containing melanocytes was different from trunk/fur-bearing skin, and more suitable for research. **b** Ear slices from the mice shown in **a** were subjected to Fontana-Masson staining for the analysis of melanin production. Scale bar = 20 μm. The relative areas positive for melanin per whole epidermis were statistically analyzed. **c** Photograph of 3D-HSSs pre-treated with UVB were incubated with or without hAMSC-CM. **d** The intensity of pigmentation in 3D-HSSs were measured by a colorimeter on the eighth day and were expressed as the L* values. Values represent the average of three samples (mean ± SD). **e** Skin tissues from the 3D-HSSs shown in (**c**) were subjected to Fontana-Masson staining for the analysis of melanin content. Scale bar = 50 μm. The relative areas positive for melanin per whole epidermis were statistically analyzed. **f** Representative histological images of LC3B expression in skin tissues from the 3D-HSSs shown in **c**. Scale bar = 50 μm. The relative areas positive for LC3II were statistically analyzed. Data are presented as mean ± SD. The experiments were repeated three times independently and the data of one representative experiment was shown. *p < 0.05; **p < 0.01, ***p < 0.001

From the above results, the hypopigmentation effect of hAMSCs was almost equivalent to hAESCs, even more obvious. Furthermore, the clinical use of stem cells requires the availability of a large number of functionally competent cells with stable phenotype, therefore hAMSCs become an attractive option due to their advantages of having higher proliferative capability and long-term survival [31]. Next, we explored the underlying molecular mechanism of hAMSC-CM-mediated hypopigmentation effect. First, C57BL/6 mice were subjected to UV for two weeks, and then, were treated with normal medium and hAMSC-CM, respectively. Since mouse ears contains epidermal melanocytes [32], the efficacy of hAMSC-CM on UVB-induced hyperpigmentation was evaluated with mouse ears. As shown in Fig. 4a, a visible tanning of ears was observed in the ears of the mice treated with UVB, and the UVB-induced alterations of pigmentation were almost disappeared in the mice treated with hAMSC-CM. The results were further confirmed by the histological examination that the fluctuations in melanin content was detected by Fontana-Masson staining (Fig. 4b).

Furthermore, we evaluated the effects of hAMSC-CM on skin lightening in vitro using 3D-HSSs (three-dimensional human skin substitutes) model, a reconstituted epidermis model containing human keratinocytes and melanocytes. UVB-induced skin darkening and melanin production were observed in the tissues of 3D-HSSs model, while hAMSC-CM treatment led to macroscopic skin lightening (Fig. 4c) and an elevated L* value (Fig. 4d) by preventing the UVB injury in the tissues of the model. In addition, the Fontana-Masson staining illuminated a significant decrease in melanin contents throughout the epidermis upon treatment with hAMSC-CM (Fig. 4e) and accompanied by the increase of autophagy-induced LC3B protein in the tissue (Fig. 4f). These studies demonstrated that hAMSC-CM efficiently protected the UVB irradiation-induced hyperpigmentation effects in mouse skin and human skin substitutes.

### hAMSCs-derived exosomes reduced α-MSH-induced hyperpigmentation

**Fig. 5:**
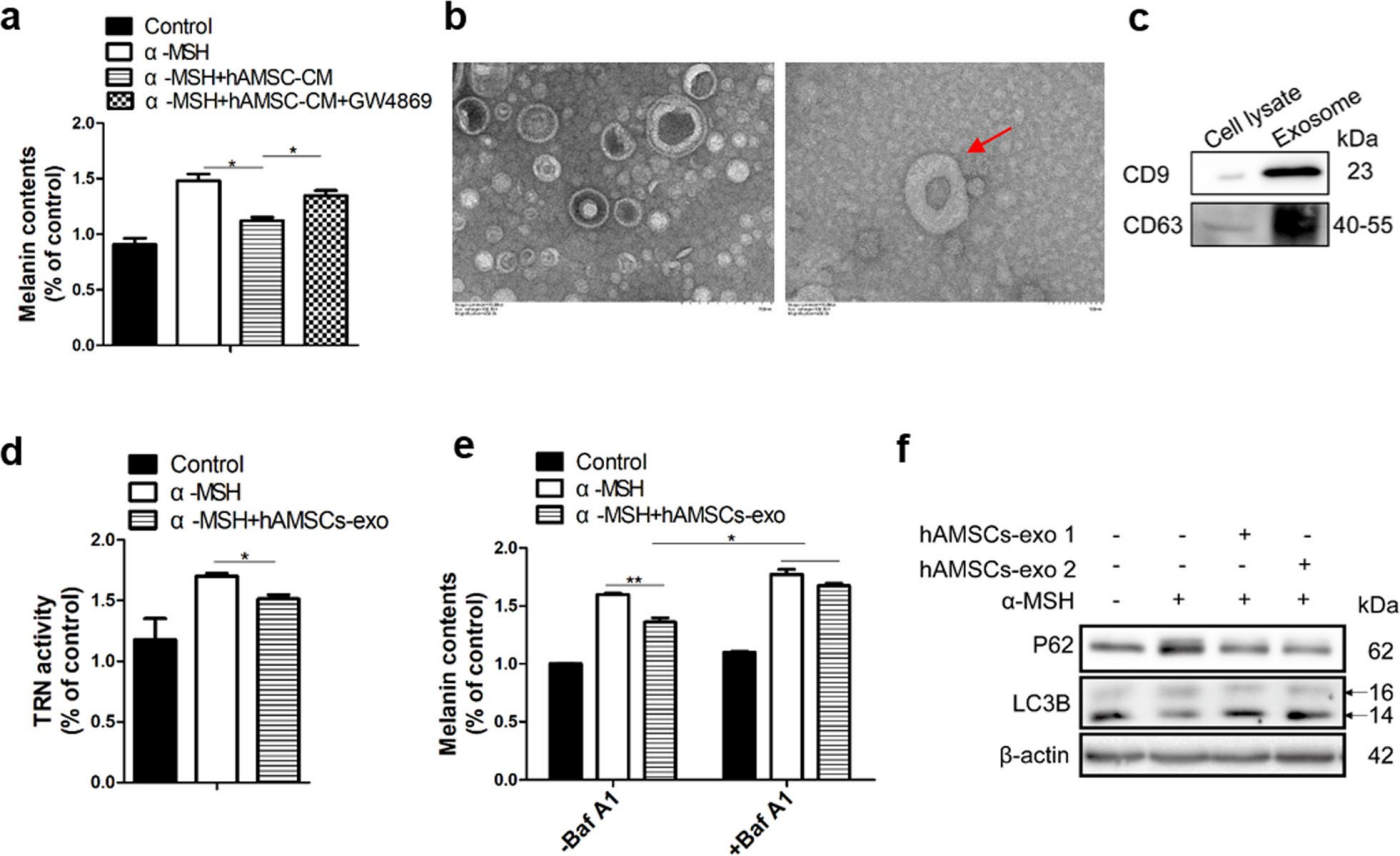
Exosomes derived from hAMSCs inhibited pigmentation by regulating autophagy. B16F10 cells were stimulated with α-MSH and the melanin contents were detected by adding hAMSC-CM that was obtained from hAMSCs treated with or without GW4869 (**a**). Morphology of exosomes under transmission electron microscopy. Left: magnification =  × 60.0 k, scale bar = 100 nm. Right: magnification =  × 50.0 k, scale bar = 200 nm (**b**). The expressions of CD63 and CD9 were analyzed by western blotting in hAMSCs-exo (**c**). Tyrosinase activity was analyzed by treatment with exosomes derived from hAMSCs (**d**). Melanin content was measured by treatment with exosomes derived from hAMSCs with or without Baf A1 (**e**). LC3B and P62 were detected by western blotting in B16F10 cells treated with hAMSCs-derived exosomes (**f**). Data are presented as mean ± SD. The experiments were repeated three times independently and the data of one representative experiment was shown. *p < 0.05; **p < 0.01, ***p < 0.001

We demonstrated that the hypopigmentation of hAMSC-CM was involved in down-regulaing melanogenesis and promoting melanosome degradation by autophagy induction. Since hAMSC-CM mainly contains cellular factors and exosomes and the potential applications of stem cells based on extracellular vesicles, the hAMSCs-derived exosomes may play a role in their hypopigmentation. Therefore, the effects of the stem cells-derived exosomes on pigmentation were determined. As showed in Fig. 5a, the hypopigmentation effect of hAMSC-CM was able to be reversed by GW4869, an exosome inhibitor, suggesting that hAMSC-derived exosomes may play a pivotal role in their hypopigmentation. Furthermore, the exosomal uptake was confirmed by monitoring PKH26 fluorescence in B16F10 cells that had been co-cultured with PKH26-labeled hAMSCs for 24 h (Additional file 1: Fig. S2a).

To assess whether hAMSCs-derived exosomes were involved in their hypopigmentation, the melanin content was determined with the exosomes. As showed in Fig. 5b–c, the morphology of the exosomes and the expression levels of the exosomal protein markers CD63 and CD9 were confirmed by transmission electron micrography and western blot analysis, respectively. Consistent with the results from hAMSC-CM, exosomes derived from hAMSCs also effectively suppressed tyrosinase activity (Fig. 5d) and cellular melanin contents (Fig. 5e). The similar results were obtained by exosomes derived from hAESCs (Additional file 1: Fig. S2b-S2d). Furthermore, the suppression of p62 protein, a known autophagy substrate, and the induction of LC3II protein were observed when B16F10 cells treated with the exosomes derived from hAMSCs (Fig. 5f). Importantly, the reduction of the cellular melanin contents was rescued by treatment with bafilomycin A1, an autophagy inhibitor (Fig. 5e). From these results, we demonstrated that hAMSCs-secreted exosomes significantly reduced the α-MSH-induced hyperpigmentation by inhibiting melanin synthesis and activating autophagy in B16F10 cells.

### Exosomal miR-181a-5p down-regulated melanin synthesis and miR-199a promoted melanosome degradation by induction of autophagy

The importance of miRNAs as key mediators of intercellular communication and potential candidates for therapy has been recently recognized. It has been demonstrated that the secreted miRNAs, especially those in extracellular vesicles (EVs) such as exosomes, serve as an intermediary to interfere with the translational machinery through binding to complementary target sequences of mRNA [33] and can be exported or imported by cells [34]. Therefore, we speculated that the hAMSCs-derived exosomes-mediated inhibition of melanin synthesis and melanosome degradation might be mainly or partially associated with their exosomal miRNAs.

**Fig. 6:**
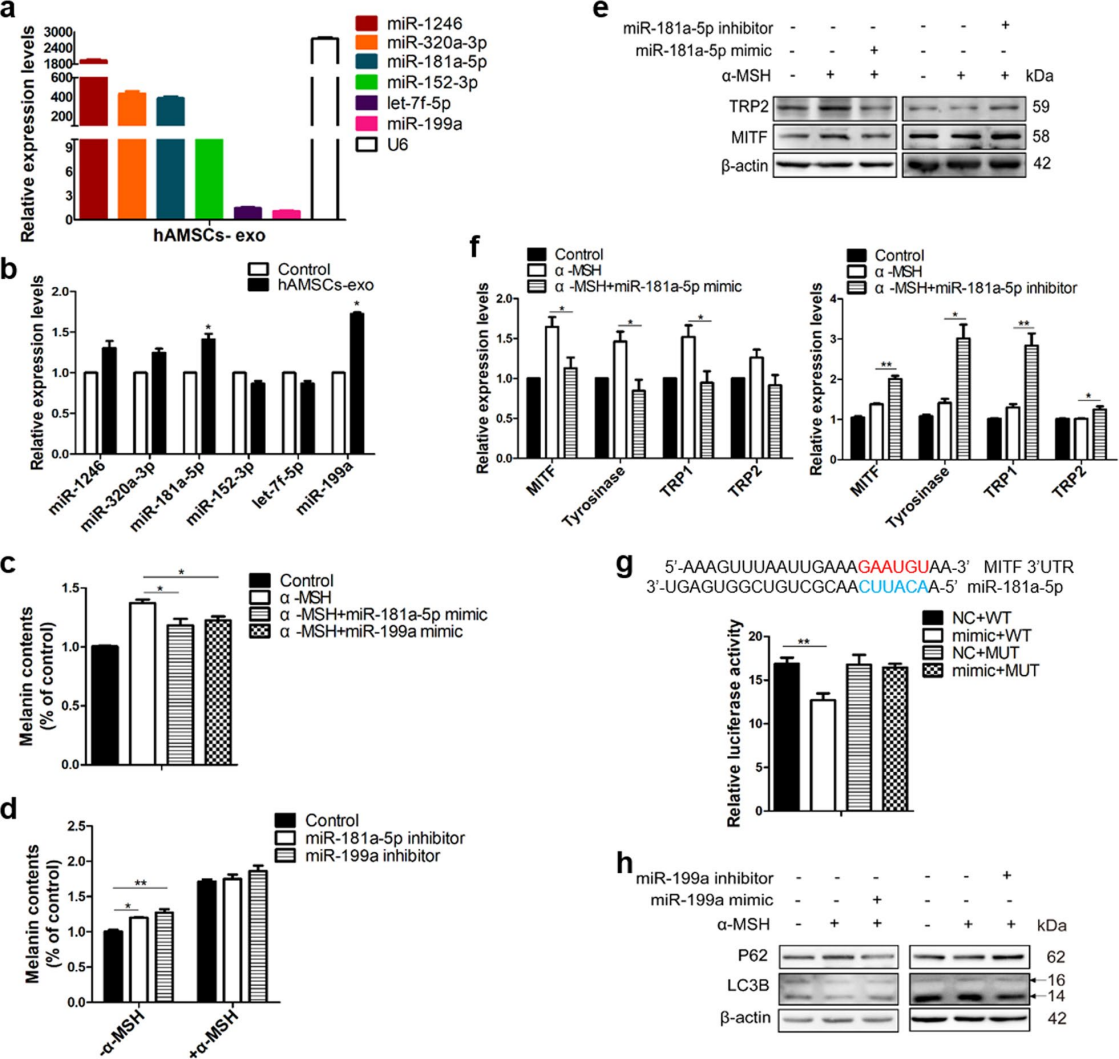
miR-181a-5p and miR-199a inhibited melanin synthesis and promoted melanosome degradation, respectively. The main miRNA contents were confirmed by RT-PCR in the exosomes obtained from hAMSCs (**a**). The expression levels of cellular miRNA were quantitated by RT-PCR, showing that miR-181a-5p and miR-199a were highly expressed in B16F10 cells treated with hAMSCs-derived exosomes (**b**). Melanin contents were measured after the transfection of the miR-181a-5p and miR-199a mimic or inhibitor into the B16F10 cells with or without α-MSH stimulation (**c**, **d**). The expression levels of the MITF, TRP2 and Tyr were measured by western blot analysis (**e**) and qRT-PCR (**f**), respectively. Dual-luciferase reporter assay showed that the relative luciferase activity which was normalized to Renilla luciferase activity was inhibited after the co-transfection of 293 T cells with the miR-181a-5p mimic and the activities were not affected by pmirGLO vector containing the WT or MUT MITF 3′-UTR (**g**). The LC3B and P62 were analyzed by western blot after the transfection of miR-199a mimic or inhibitor (**h**). Data are presented as mean ± SD. The experiments were repeated three times independently and the data of one representative experiment was shown. *p < 0.05; **p < 0.01, ***p < 0.001

To further determine the components of the exosomes that might be responsible for regulating pigmentation, 781 exosomal miRNAs were identified by deep sequencing in hAMSCs-secreted exosomes (Additional file 1: Table S2) and the analysis of miRNA spatial and temporal expression patterns suggested that 23 exosomal miRNAs might be the potential regulators of skin pigmentation from miRNA expression profile. Next, we integrated different bioinformatics servers of miRNA-target prediction and functional enrichment analysis to identify 6 miRNAs (miR-1246, miR-152-3p, miR-181a-5p, miR-320a-3p, let-7f-5p, miR-199a) for further investigation (Additional file 1: Fig. S3a-S3c). The 6 miRNAs described above were further confirmed by real-time PCR analysis in hAMSCs-exo (Fig. 6a), in which miR-181a-5p and miR-199a were prominently increased during a 24h period in B16F10 treated with hAMSCs-exo (Fig. 6b).

In addition, a significant hypopigmentation was observed in B16F10 cells treated with miR-181a-5p mimic or miR-199a mimic (Fig. 6c). In contract, miR-181a-5p inhibitor or miR-199a inhibitor had no effects on melanin synthesis with α-MSH stimulation or moderately increased the cellular melanin contents without α-MSH stimulation in B16F10 cells (Fig. 6d). In addition, miR-181a-5p mimic led to a substantial decrease in the mRNA and protein expressions of MITF were observed in B16F10 cells with α-MSH challenge, resulting in the repressions of the transcriptions for its downstream genes such as tyrosinase, TRP1, TRP2 (Fig. 6e, f). Conversely, silencing miR-181a-5p significantly increased MITF expressions in B16F10 (Fig. 6e, f), indicating that MITF is a key protein for melanin synthesis. Bioinformatics analysis predicted that miR-181a-5p directly regulates expression of MITF by targeting its 3’UTR. The alignments between miR-181a-5p and 3’UTR of MITF represented putative target sequences which can confer inhibition of translation by miR-181a-5p (Fig. 6g). Furthermore, we introduced sequences of the wild-type or mutated 3’UTR of MITF into the pmirGLO luciferase reporter vector. Luciferase reporter assay showed that miR-181a-5p mimic significantly repressed pmirGLO-WT-3’UTR luciferase activity, while the mutation of the miR-181a-5p binding site abolished the inhibition (Fig. 6g). These results demonstrated that miR-181a-5p directly targeted MITF to inhibit melanogenesis, suggesting that miR-181a-5p might be a potential anti-melanogenic candidate.

Next, we evaluated the role of miR-199a in melanosome degradation, and the results showed that miR-199a promoted autophagy and accompanied by accumulation of LC3II and consumption of p62/SQSTM1 (Fig. 6h). Opposite results were observed in the cells treated with miR-199a inhibitor. Recent studies revealed that miR-199a played a role in mediating autophagy through targeting mTOR [35]. Therefore, our results suggested that miR-199a might regulate melanosome degradation by activating autophagy. All these results pointed out that the hypopigmentation effect of the exosomes derived from hAMSCs may be at least partially mediated by the exosomes-derived miR-181a-5p and miR-199a. Whether other miRNA derived from hAMSCs exosomes contribute to the synergistic acceleration of hypopigmentation need to be further investigated in the future.

## Discussion

Melanin is synthesized in melanosome, a specialized cellular organelle of melanocytes and then transported to adjacent keratinocytes in epidermis. Although melanin is important to protect human skin against harmful UVB injury, toxic chemicals and other environment factors, the abnormal accumulation of melanin lead to hyperpigmentation and dermatological problems such as freckles, age spots and melasma in skin [36]. Human skin pigmentation depends on intra- and extracellular factors including genetic background, and is highly regulated and maintained by multiple metabolic pathways and signaling networks.

Currently, anti-melanogenic agents that target tyrosinase activity, melanosome maturation or melanogenesis-related signaling pathways have been studied [37]. However, numerous anti-melanogenic candidates have been limited in application due to a lack of efficacy or adverse side effects [38, 39]. New therapeutic modalities of hyperpigmented disorders in skin has been a long-term goal for cosmetic and pharmaceutical applications.

In recent years, stem cells have been reported to have therapeutic potential in various diseases including diabetes, cancer, skin injury and neural disorders, etc. In the application of stem cells, the potential therapies based on extracellular vesicle deserve attention due to their versatility. Exosomes are small membrane vesicles released by most cells including stem cells, and are involved in intercellular communication and progression of diseases such as cancer, through the delivery of messenger RNAs (mRNAs), miRNAs, proteins, etc. Although stem cell therapy has become a very promising and advanced approach, there are still many obstacles that need to be overcome in the future. We and others reported that mesenchymal stem cells and their conditional medium promote cutaneous wound healing by accelerating wound closure, promoting cell proliferation and inhibiting cell apoptosis through a paracrine manner [16–18, 24]. However, the effect and mechanism of human amniotic stem cells on skin hyperpigmentation is still explored.

In the present study, we demonstrated for the first-time the anti-melanogenesis of hASCs and their CM in skin pigmentation. We provided strong evidence that hASCs and their conditional medium including hAMSC-CM or hAESC-CM significantly inhibited melanin synthesis, tyrosinase activity, and the expressions of MITF, TRP1, tyrosinase. Unlike a reported anti-melanogenic agent kojic acid with tumorigenic activity, human amniotic stem cells have multiple advantages for clinical applications, such as low immunogenicity, no tumorigenicity, multipotency, without ethical concerns and the beneficial paracrine effects including inmmunosuppressive and imnnunoregulative effects and have been considered a promising source of seed cells for biological therapeutics [40]. Overall, human amniotic stem cell conditional medium is a novel and relatively safe whitening agent.

Although the precise mechanism underlying skin pigmentation has not been fully elucidated, three important processes which are involved in skin pigmentation have been identified: melanin synthesis in melanocytes [41], melanosomes are transported to surrounding keratinocytes [42], and melanosomes degradation [43]. Upon exposure to UVB, a variety of paracrine cytokines trigger melanogenesis through regulating various signaling pathways, including α-MSH/cAMP-dependent signaling pathway, ERK and p38/MAPK signaling pathway, autophagy signaling pathway, PI3K/Akt signaling pathway, nitric oxide signaling pathway, melanosome transfer mechanism such as exocytosis, fusion of plasma membranes and transfer by membrane vesicles [44, 45]. Our results indicated that autophagy was critically required for melanosomes degradation in hAMSC-CM or hAESC-CM-treated cells. In line with this, a previous study also showed autophagy played a significant role in determining skin color by regulating melanosome degradation in keratinocytes and melanocytes [11–13]. However, it is believed that heteropolymeric melanin produced in melanosomes required harsh conditions for degradation. As autophagy is argued to be an indirect mode of cellular clearance by selectively targeting harmful materials [46], therefore, it is reasonable to speculate that autophagy selectively removes abnormal accumulation of melanosomes to protect cells from the toxicity induced by harmful factors such as UV or chemical exposure. This may explain why hASCs and their CM exhibit a potent inhibitory effect on melanin deposition caused by external stimuli, such as α-MSH or UVB. Therefore, hASCs-derived CM reduced excessive melanin deposition by coordinating functionality of melanogenesis and melanosome degradation to maintain skin physiological homeostasis.

Compared with hAESCs, our results established that hAMSCs were more conducive to their applications due to their high reproductive capability, long-term survival and more obvious effects. Therefore, hAMSC-CM was used to further investigate their components that were responsible for regulating pigmentation. We demonstrated that hAMSC-CM was an effective inhibitor of hyperpigmentation in the experiments of mouse models and 3D-HSSs. We previously observed that hAMSCs-derived conditional medium play a key role in cutaneous wound healing through their paracrine effects [24]. However, the effects of hAMSCs-derived exosomes on skin pigmentation has not yet to be explored. Intriguingly, we observed a significant whitening phenotype in B16F10 cells treated with hAMSC-secreted exosomes, in which the decreases in the cellular melanin contents were restored by treatment with bafilomycin A1, an autophagy inhibitor, suggesting that autophagy might be involved in their hypopigmentation. All these results demonstrated that hAMSCs-secreted exosomes might play a central role in modulating α-MSH-induced hyperpigmentation.

Given the fact that hASCs-derived exosomes possessed an effective hypopigmentation effect, we speculated that the underlying mechanism might be involved in some components of their exosomes. Although exosomes contain various molecules from parental cells, miRNA is the most well-studied molecule type in exosome because of its important functions and biomarker properties. Thus, the exosomal miRNAs were evaluated by deep sequencing. We identified that the anti-melanogenesis effect of miR-181a-5p was similar to that of exosomes which repressed the mRNA and protein expression of MITF in B16F10 cells treated with α-MSH. Moreover, miR-181a-5p directly regulates expression of MITF by targeting its 3’UTR, causing the inhibition of the transcriptions for its downstream melanogenesis-related genes (tyrosinase, TRP1 and TRP2). Furthermore, we also demonstrated that the significant hypopigmentation of miR-199a may be related to the activation of autophagy through the targeted inhibition of mTOR, which eventually led to the melanosomes degradation. Although the importance of the exosomal miRNA in inhibitory effect on hyperpigmentation has been verified, we were not able to exclude the moderate inhibitory effect of other factors in exosomes, such as mRNA, lncRNA, circRNA and proteins. Moreover, a specific miRNA may target numerous proteins, whereas a protein may be also modulated by several miRNAs, making the effects of miRNAs more complicated. Therefore, our results might partly explain why hASCs-derived exosomes possess a potent hypopigmentation.

## Conclusion

**Fig. 7:**
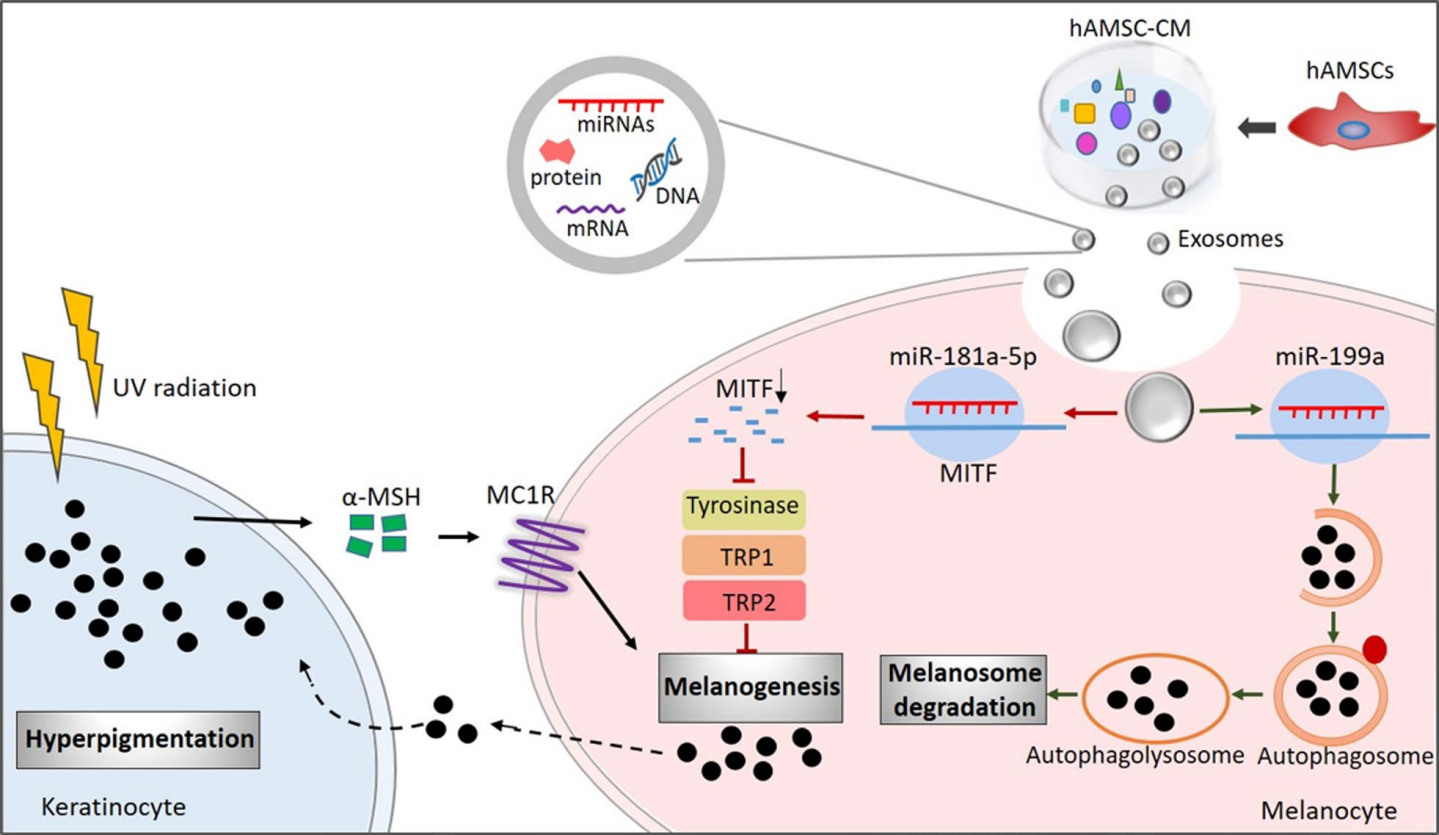
A model of the anti-hyperpigmentation of hAMSC-CM. Upon UV radiation, keratinocytes secrete α-MSH to induce MITF expression and promote melanogenesis in melanocyte. hAMSC-CM and hAMSCs-derived exosomes reduce excessive melanin deposition by suppressing melanogenesis and promoting melanosome degradation. Furthermore, exosomes-derived miR-181a-5p and miR-199a reduce MITF expression to suppress melanin content and regulating autophagy flux to promote melanosome degradation, respectively

Although it has been reported that human umbilical cord blood (hUCB)-MSCs have an anti-melanogenesis effect [47], the molecular mechanism remains unclear. In the present study, we demonstrated that human amniotic stem cells and their conditional medium significantly inhibited α-MSH or UVB-induced skin hyperpigmentation through suppressing melanogenesis and promoting melanosome degradation in vivo and in vitro. Furthermore, we identified that hASCs exosomes-derived miR-181a-5p and miR-199a were responsible for their anti-hyperpigmentation by reducing MITF expression to suppress melanogenesis and activating autophagy to promote melanosome degradation, respectively (Fig. 7). Therefore, our data demonstrated that hASCs-exo or miR-181a-5p and miR-199a might serve as an important mediator and novel potential candidate for therapy of skin hyperpigmentation clinically.

## Supplementary Information

**Additional file 1**. **Figure S1**. The identification of hASCs and the effect of their CM on the proliferation and migration of B16F10. **Figure S2**. Exosomes derived from hAESCs inhibited hyperpigmentation. **Figure S3**. Bioinformatics analysis of hAMSCs-derived exosomes. Figure S4. Quantitative analysis is shown in Western blotting. **Table S1**. The sequences of Real-Time PCR primers. **Table S2**. miRNAs expression in top from hAMSCs-secreted exosomes.

## Data Availability

The data that support the findings of this study are available from the corresponding author upon reasonable request.

## Abbreviations

hASCs: Human amniotic stem cells
hAMSCs: Human amniotic mesenchymal stem cells
hAESCs: Human amniotic epithelial stem cells
hAMSC-CM: hAMSC conditional medium
hAESC-CM: hAESC conditional medium
α-MSH: α-Melanocyte-stimulating hormone
MITF: Microphthalmia-associated transcription factor
TRP1: Tyrosinase-related protein 1
3D-HSSs: Three-dimensional human skin substitutes
