# Supplementary material for: Human amniotic stem cells-derived exosmal miR-181a-5p and miR-199a inhibit melanogenesis and promote melanosome degradation in skin hyperpigmentation, respectively
Source: Stem Cell Res Ther. 2021 Sep 10;12:501. doi: 10.1186/s13287-021-02570-9 (PMC8431893; doi:10.1186/s13287-021-02570-9)
Supplement: Supplementary file 1 — Additional file 1. Figure S1. The identification of hASCs and the effect of their CM on the proliferation and migration of B16F10. Figure S2. Exosomes derived from hAESCs inhibited hyperpigmentation. Figure S3. Bioinformatics analysis of hAMSCs-derived exosomes. Figure S4. Quantitative analysis is shown in Western blotting. Table S1. The sequences of Real-Time PCR primers. Table S2. miRNAs expression in top from hAMSCs-secreted exosomes. [file 13287_2021_2570_MOESM1_ESM.docx]

Supplementary Materials for

Human amniotic stem cells-derived exosmal miR-181a-5p and miR-199a inhibit melanogenesis and promote melanosome degradation in skin hyperpigmentation, respectively

Xiao-Yu Wang, Xiao-Hui Guan, Zhen-Ping Yu, Jie Wu, Qi-Ming Huang, Ke-Yu Deng^*^ and Hong-Bo Xin^*^

Correspondence to: xinhb@ncu.edu.cn;

**This PDF file includes:**

Figures. S1 to S4

Table. S1 to S2


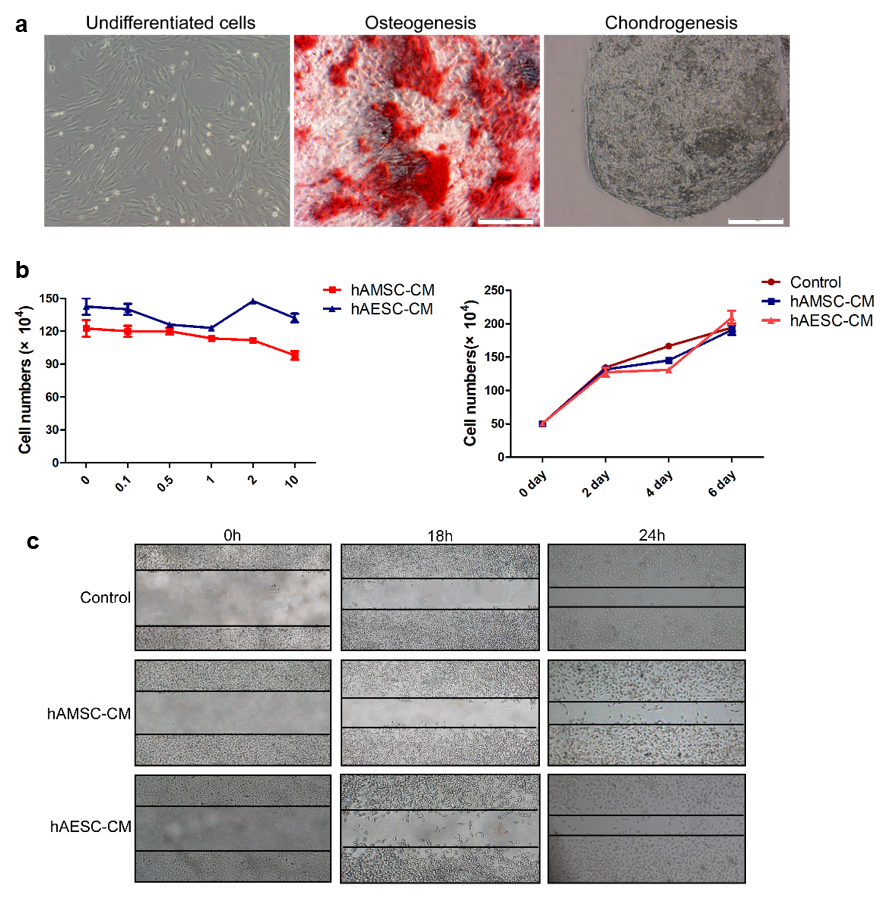


Figure. S1.

**The identification of hASCs and the effect of their CM on the proliferation and** [**migration**](../../../../Dict/8.5.3.0/resultui/html/index.html#/javascript:;) **of B16F10.**

**(a)** Multiple differentiation ability of hAMSCs. Osteocytes and chondrocytes differentiated from hAMSCs were determined by staining with Alizarin Red and Alcian Blue, respectively. **(b)** B16F10 cells were treated with various concentrations of hAMSC-CM or hAESC-CM (left) and treated with 2×10^5^ cells/mL concentration of hAMSC-CM or hAESC-CM at different time points (right), then cell numbers were counted. ‘1’ represents 2×10^5^ cells/mL concentration. **(c)** Cell migration was quantified by wound strack assay treated with normal medium, hAMSC-CM and hAESC-CM for 18 h or 24 h. The experiments were repeated three times independently and the data of one representative experiment was shown.


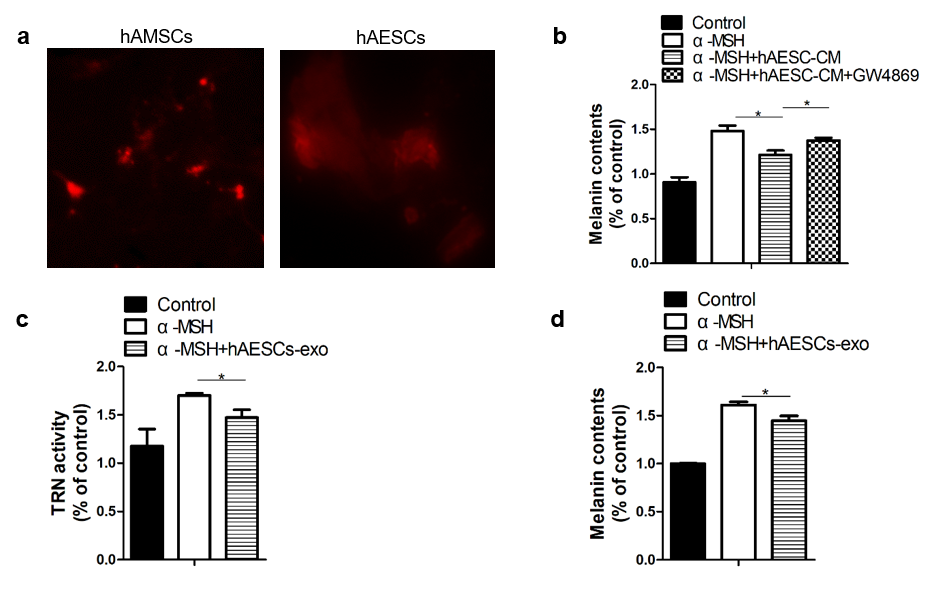


Figure. S2.

**Exosomes derived from hAESCs inhibited hyperpigmentation.**

(**a**) Representative images of PKH26-labled exosomes (red) in hAMSCs and hAESCs. (**b**) B16F10 cells were stimulated with α-MSH and melanin content was detected by treatment with hAESC-CM in the presence or absence of GW4869. (**c**) Tyrosinase activity was analyzed by treatment with exosomes derived from hAESCs. (**d**) Melanin content was measured by treatment with exosomes derived from hAESCs. The experiments were repeated three times independently and the data of one representative experiment was shown.

**
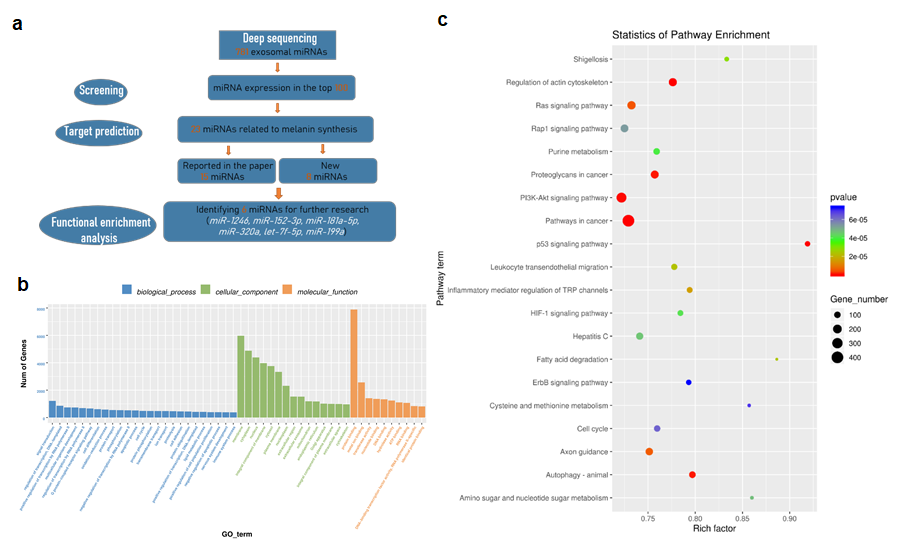
**

Figure. S3.

**Bioinformatics analysis of hAMSCs-derived exosomes.**

(**a**) Identifying 6 miRNAs for further research by bioinformatics analysis and literature review. (**b**) GO enrichment scatterplot. (**c**) KEGG enrichment pathway.


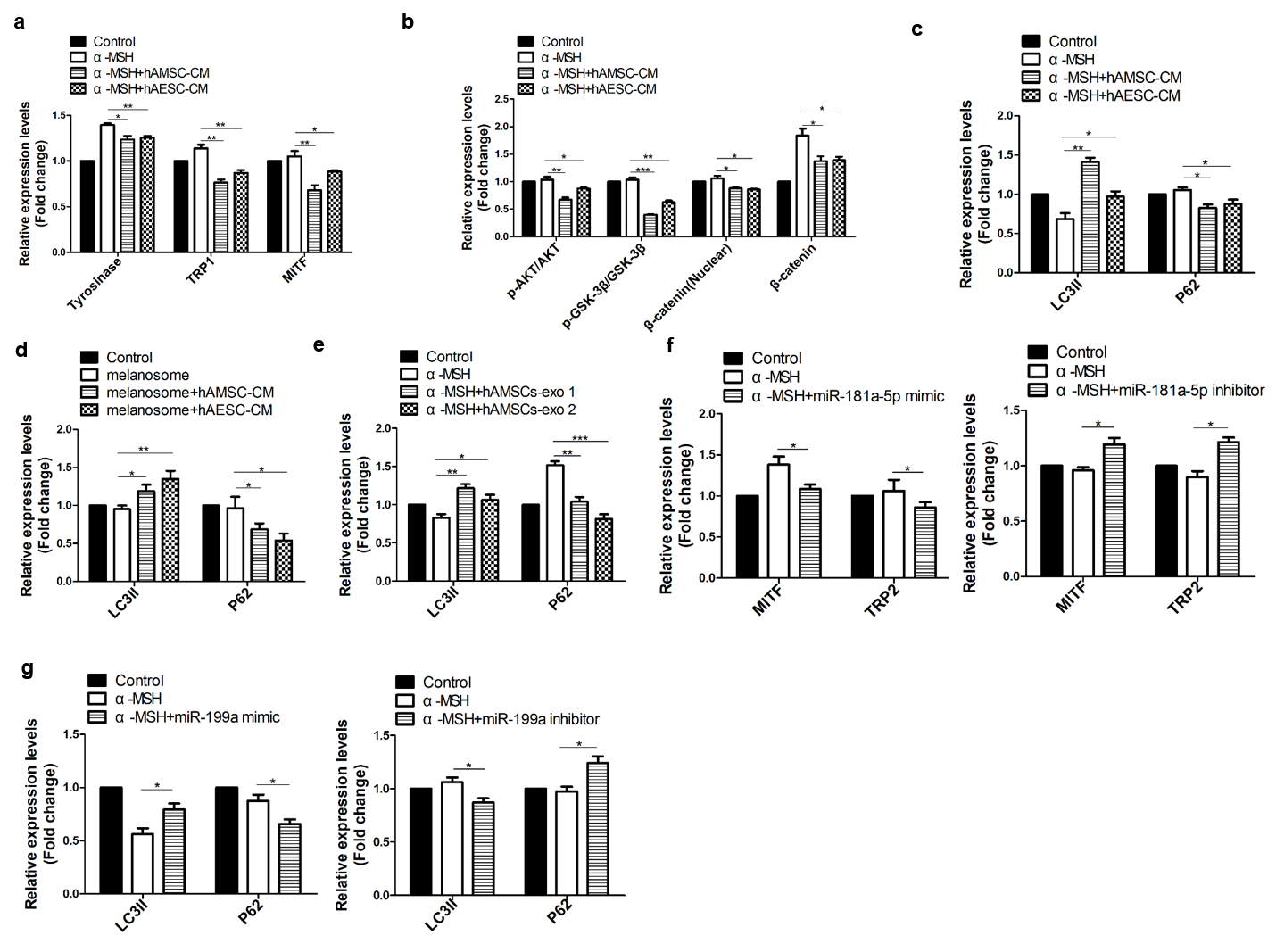
Figure. S4.

**Quantitative analysis is shown in Western blotting**

(**a**) Relative expression of tyrosinase, TRP1 and MITF were quantified in Fig. 2k. (**b**) Relative expression of p-AKT/AKT, p-GSK-3β/GSK-3β, β-catenin (nuclear)/PCNA and β-catenin were quantified in Fig. 2m. (**c**) Relative expression of LC3II and P62 were quantified in Fig. 3a. (**d**) Relative expression of LC3II and P62 were quantified in Fig. 3f. (**e**) Relative expression of LC3II and P62 were quantified in Fig. 5e. (**f**) Relative expression of MITF and TRP2 were quantified in Fig. 6e. (**g**) Relative expression of LC3II and P62 were quantified in Fig. 6h. Data represents the average of three independent experiments (mean ± SD). The data were normalized to β-actin.

Table S1. The sequences of Real-Time PCR primers.

| ***Gene*** | ***Sequence (5' to 3')*** |
| --- | --- |
| Tyrosinase | Forward: TGACAAAGCCAAAACCCCCA |
|  | Reverse: AGCCATTGTTCAAAAATACTTCCA |
| MITF | Forward: GGGAGCTCACAGCGTGTATT |
|  | Reverse: AGCTCCTTAATGCGGTCGTT |
| TRP1 | Forward: GGGATTCATGGTACTGCTGTATT |
|  | Reverse: AGGGGGAGGACGTTGTAAGA |
| TRP2 | Forward: GGACATGCAAATGCACAGGAAA |
|  | Reverse: GTCTAAGGCGCCCAAGAACT |
| GAPDH | Forward: AGCCAAAAGGGTCATCATCT |
|  | Reverse: GGGGCCATCCACAGTCTTCT |

**Table S2. miRNAs expression in top from hAMSCs-Secreted Exosomes.**

| ***Index*** | ***Reporter Name*** | ***Target Sequence*** ***(5' to 3')*** |
| --- | --- | --- |
| 1 | hsa-let-7f-5p | TGAGGTAGTAGATTGTATAGTT |
| 2 | hsa-let-7i-5p | TGAGGTAGTAGTTTGTGCTGTT |
| 3 | hsa-let-7g-5p | TGAGGTAGTAGTTTGTACAGTT |
| 4 | hsa-miR-10a-5p_R-1 | TACCCTGTAGATCCGAATTTGT |
| 5 | hsa-miR-20a-5p | TAAAGTGCTTATAGTGCAGGTAG |
| 6 | hsa-miR-21-5p_R+1 | TAGCTTATCAGACTGATGTTGAC |
| 7 | hsa-miR-22-3p | AAGCTGCCAGTTGAAGAACTGT |
| 8 | hsa-miR-23a-3p_R+1 | ATCACATTGCCAGGGATTTCCA |
| 9 | hsa-miR-26b-5p_R+1 | TTCAAGTAATTCAGGATAGGTT |
| 10 | hsa-miR-26a-5p | TTCAAGTAATCCAGGATAGGCT |
| 11 | hsa-miR-27a-3p_R-1 | TTCACAGTGGCTAAGTTCCG |
| 12 | hsa-miR-27b-3p | TTCACAGTGGCTAAGTTCTGC |
| 13 | hsa-miR-29a-3p | TAGCACCATCTGAAATCGGTTA |
| 14 | hsa-miR-30a-5p_R+2 | TGTAAACATCCTCGACTGGAAGCT |
| 15 | hsa-miR-99b-5p | CACCCGTAGAACCGACCTTGCG |
| 16 | hsa-miR-100-5p | AACCCGTAGATCCGAACTTGTG |
| 17 | hsa-miR-125b-5p | TCCCTGAGACCCTAACTTGTGA |
| 18 | hsa-miR-127-3p | TCGGATCCGTCTGAGCTTGGCT |
| 19 | hsa-miR-143-3p_R+1 | TGAGATGAAGCACTGTAGCTCT |
| 20 | hsa-miR-146a-5p | TGAGAACTGAATTCCATGGGTT |
| 21 | hsa-miR-148a-3p | TCAGTGCACTACAGAACTTTGT |
| 22 | hsa-miR-151b_R+3 | TCGAGGAGCTCACAGTCTAGT |
| 23 | hsa-miR-152-3p | TCAGTGCATGACAGAACTTGG |
| 24 | hsa-miR-181a-5p | AACATTCAACGCTGTCGGTGAGT |
| 25 | hsa-miR-191-5p | CAACGGAATCCCAAAAGCAGCTG |
| 26 | hsa-miR-199a-5p | CCCAGTGTTCAGACTACCTGTTC |
| 27 | hsa-miR-199a-3p_R-1 | ACAGTAGTCTGCACATTGGTT |
| 28 | hsa-miR-199b-5p | CCCAGTGTTTAGACTATCTGTT |
| 29 | hsa-miR-221-3p | AGCTACATTGTCTGCTGGGTTTC |
| 30 | hsa-miR-222-3p_R+3 | AGCTACATCTGGCTACTGGGTCTC |
| 31 | hsa-miR-320a-3p | AAAAGCTGGGTTGAGAGGGCGA |
| 32 | hsa-miR-335-5p | TCAAGAGCAATAACGAAAAATGT |
| 33 | hsa-miR-423-3p | AGCTCGGTCTGAGGCCCCTCAGT |
| 34 | hsa-miR-424-5p_R-1 | CAGCAGCAATTCATGTTTTGA |
| 35 | hsa-miR-503-5p_R-1 | TAGCAGCGGGAACAGTTCTGCA |
| 36 | hsa-miR-1246_R+1 | AATGGATTTTTGGAGCAGGG |
| 37 | hsa-miR-3195_L+3R-1 | CGGCGCGCCGGGCCCGGGT |
| 38 | hsa-mir-7110-p3_1ss1TC | CTCTCTCTCTCTCTCTCCC |
| 39 | hsa-miR-12136_R+8 | GAAAAAGTCATGGAGGCCATGGGGTT |
| 40 | ssc-mir-1285-p5_1ss17TC | CTGTGAATAGCCACTGCACTC |
| 41 | ssc-miR-1285_L+1R-5 | CCTGGGCAACATAGCGAGAC |
| 42 | bta-mir-2904-2-p5 | GAGCCTCGGTTGGCCTCGGA |
| 43 | bta-mir-2904-2-p3_1ss1CT | TGTCCCCGCCGGCGGGCC |
| 44 | mmu-mir-5119-p3_1ss1GC | CTCATCTCATCCTGGGGC |
| 45 | bta-miR-1246_L-1R+2 | ATGGATTTTTGGAGCAGGGA |
| 46 | mmu-miR-2137_L-2R-1_1ss16AG | CGGCGGGAGCCCCGGGGA |
| 47 | mmu-miR-2137_L-2_1ss16AG | CGGCGGGAGCCCCGGGGAG |
| 48 | bta-mir-2887-1-p5_1ss21AT | GACCGGGGTCCGGTGCGGAGTGC |
| 49 | bta-mir-2887-1-p3_1ss11AT | CGGTGCGGAGTGCCCTTC |
| 50 | bta-mir-2887-1-p5_1ss20AT | ACCGGGGTCCGGTGCGGAGTGC |
| 51 | bta-mir-2887-1-p3_1ss21AT | GACCGGGGTCCGGTGCGGAGTGC |
| 52 | mmu-miR-5106_R-4_1ss1AG | GGGTCTGTAGCTCAGTTGG |
| 53 | mmu-mir-6236-p5_1 | AAAATGGATGGCGCTGGAGC |
| 54 | mmu-mir-6236-p3_1ss23GC | AAAATGGATGGCGCTGGAGCGTCGGG |
| 55 | mmu-mir-6236-p5_2 | CCTGAAAATGGATGGCGCTGGAGC |
| 56 | mmu-mir-6236-p3_1ss20GC | ATGGATGGCGCTGGAGCGTCGGG |
| 57 | mmu-mir-6236-p3_1 | AATCAACTAGCCCTGAAAA |
| 58 | mmu-mir-6236-p3_3 | GCCCTGAAAATGGATGGC |
| 59 | mmu-mir-6236-p3_2 | CTGAAAATGGATGGCGCTGGAGC |
| 60 | mmu-mir-6240-p5_1ss13TG_2 | GCGGGTGTTGACGCGATGTGAT |
| 61 | mmu-mir-6240-p3_1ss13TG | GCGGGTGTTGACGCGATGTGATTTC |
| 62 | mmu-mir-6240-p5_1ss13TG_1 | GCGGGTGTTGACGCGATGTGA |
| 63 | mmu-mir-6240-p3_1ss9TG | GTGTTGACGCGATGTGATTTCTGC |
| 64 | mmu-mir-6240-p5_1ss20GT | TTCTGCCCAGTGCTCTGAAT |
| 65 | mmu-mir-6240-p5_2 | GTGATTTCTGCCCAGTGCTCTG |
| 66 | mmu-mir-6240-p5_1 | ATGTGATTTCTGCCCAGTGCT |
| 67 | mmu-mir-6240-p5_1ss1TG | GCGATGTGATTTCTGCCCAGT |
| 68 | bta-miR-11987_L-1_1ss8TA | GAGGAAACTCTGGTGGAGGT |
| 69 | bta-miR-11987_L-2R-1_1ss8TA | AGGAAACTCTGGTGGAGG |
| 70 | bta-miR-11987_L-2_1ss8TA | AGGAAACTCTGGTGGAGGT |
| 71 | bta-miR-11980_R-1_1ss4CG | AGGGAACGGGCTTGGCGGA |
| 72 | PC-5p-33089_123 | ATGAGGTGTGATTAGGAG |
| 73 | PC-3p-7250_510 | GTTCGACTCCCGGTATGGGAACCA |
| 74 | PC-3p-14422_286 | CAAGATGAGATCTCCCGGG |
| 75 | PC-3p-25313_165 | AGAGCACTGTTATGGCTAGG |
| 76 | PC-3p-19762_213 | GTTTTAGTGGAATGCTCT |
| 77 | PC-3p-7780_482 | CCGACCTGCACGAATGGC |
| 78 | PC-5p-5611_625 | ATGGTAGAATTCCAGGTGT |
| 79 | PC-3p-45625_83 | CGATGTCGGCTCATCTCAT |
| 80 | PC-5p-17351_241 | CCATGTGGAAAGGCACAGAC |
| 81 | PC-5p-24826_169 | TTACTCCTTGAGCTATTGG |
| 82 | PC-5p-5304_654 | AACCCGGTCAGCCCCTCTCCG |
| 83 | PC-3p-5133_670 | GTGGGAGGCTTTGAAACC |
| 84 | PC-5p-252_5286 | GTTGTATAGTCAAGTAAT |
| 85 | PC-5p-1863_1370 | CGCTAGTGTTTGTGGAGC |
| 86 | PC-3p-18136_232 | TGAGATTCCCTCAGTAGC |
| 87 | PC-3p-21094_200 | GTGAAGACGAGTAGGGCGGG |
